# Postsurgical Diagnosis of an Unusual Case of Primary Hepatic Lymphoma Presenting as Liver Abscess with an Uncommon Complication: A Hepatogastric Fistula

**DOI:** 10.1155/2021/6647558

**Published:** 2021-02-24

**Authors:** Mbarek Yaka, Farid Chehab, Mohmed Allaoui, Abdelmonaim Ait Ali, Aziz Zentar

**Affiliations:** ^1^Department of Surgery, Military University Hospital Med V, University Hassan II of Casablanca, Casablanca, Morocco; ^2^Department of Surgery, Ibn Rochd University Hospital, University Hassan II of Casablanca, Casablanca, Morocco; ^3^Department of Pathology, Military University Hospital Med V, Rabat, Morocco; ^4^Department of Surgery, Military University Hospital Med V, Rabat, Morocco

## Abstract

Primary hepatic lymphoma (PHL) is a very rare malignancy and constitutes 0.016% of all cases of non-Hodgkin's lymphoma and 0.4% of extranodal non-Hodgkin's lymphoma. We describe a rare case of primary hepatic lymphoma presenting as liver abscess which was complicated with the development of a hepatogastric fistula. A 58-year-old man presented with clinical signs of sepsis, high-grade fever, right upper abdominal pain, and weight loss which had progressed in the past 8 months. Noncontrast abdominal computed tomography (CT) revealed a heterogeneously hypodense lesion in the left lobe of the liver with multiple air foci within, which are seen to extend into the body of the stomach. The patient was initially misdiagnosed as a case of rupture of liver abscess into the stomach. Postoperative liver biopsy examination confirmed a diagnosis of diffuse large B-cell lymphoma. Systemic staging revealed no evidence of nodal or bone marrow involvement, so PHL was diagnosed. Chemotherapy was initiated, but discontinued due to the patient's general condition. Finally, the patient succumbed to neutropenic fever following chemotherapy. Here, we present the exceptional case of a primary hepatic lymphoma with an unusual complication, a hepatogastric fistula, and try through the existing literature to show the difficulties involved in diagnosis and treatment.

## 1. Introduction

PHL is a rare form of non-Hodgkin lymphoma (NHL), and it is a tumor confined to the liver without evidence of lymphomatous involvement of the lymph nodes, spleen, bone marrow, or other lymphoid structures. The vast majority of PHL is NHL, most often a diffuse large B-cell lymphoma type. PHL was often misdiagnosed as some other tumor and frequently diagnosed intra- or postoperatively. We report an interesting case of PHL that was difficult to discriminate from a liver abscess, with an unusual complication, hepatogastric fistula, diagnosed after postoperative biopsy report, and try through the existing literature to show the difficulties involved in diagnosis and treatment.

## 2. Case Report

**Figure 1:**
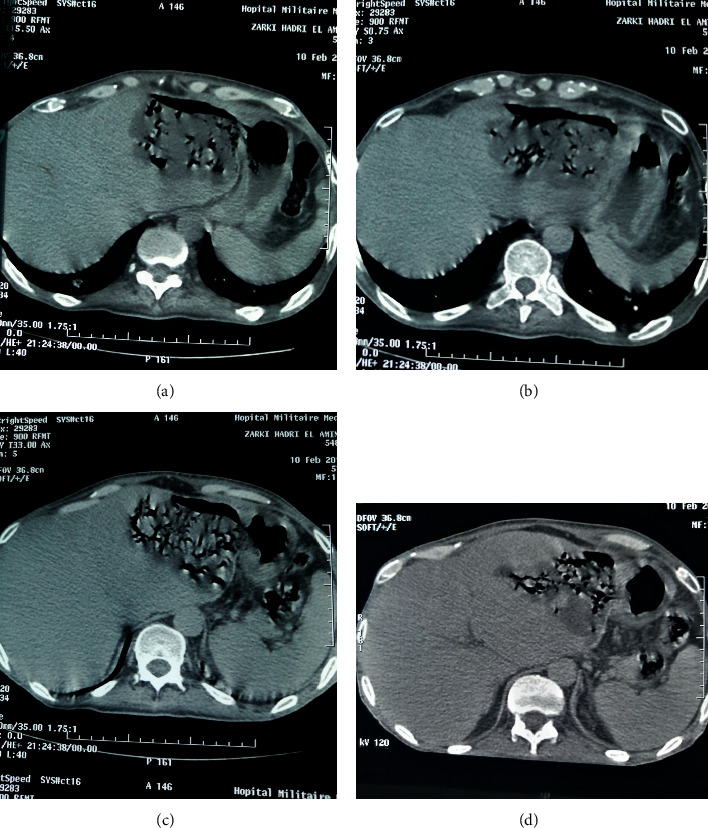
Preoperative CT scan showing (a, b) liver cystic mass containing multiple air foci; (c, d) hepatogastric fistula.

**Figure 2:**
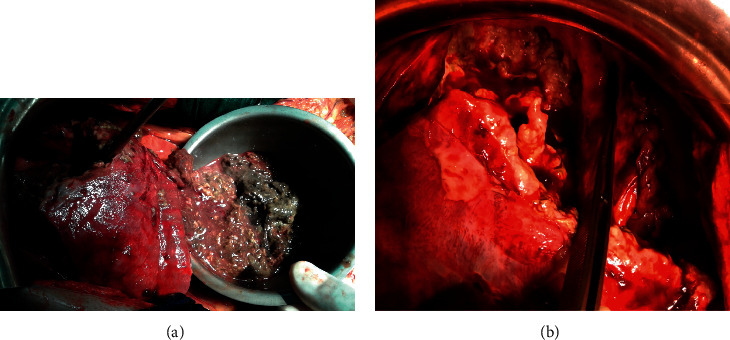
Operative view of the anterior surface of the left lobe of the liver showing (a) necrotic area of liver lymphoma and (b) hepatogastric fistula.

**Figure 3:**
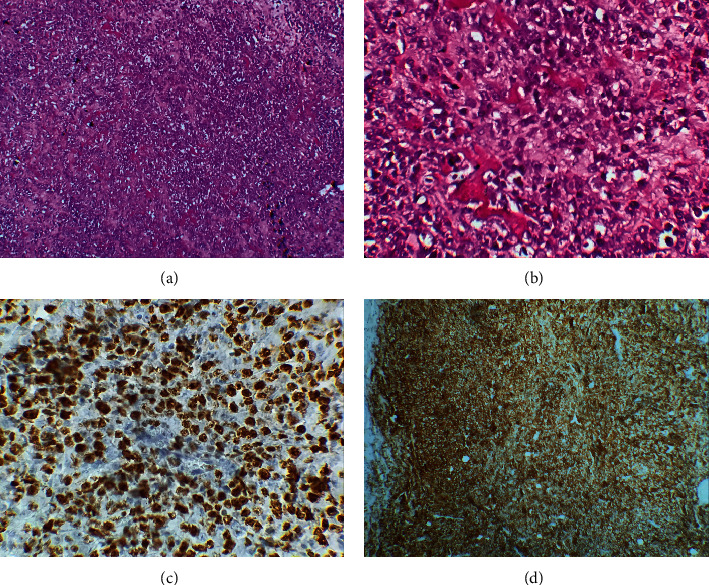
Histopathological examination. (a) Diffuse proliferation of atypical lymphoid cells (HE, GX 40). (b) Large numbers of lymphocytes ranging in size from medium to large, with oval or round nuclei containing fine chromatin and scanty cytoplasm (HE, GX 200). (c) Immunohistochemical staining was positive for CD20 (a B-cell marker). (d) The Ki-67 index was positive in 80%.

A 58-year-old male with no significant past medical history presented to our emergency department with fever, right upper abdominal pain, vomiting, hematemesis, anorexia, night sweats, and unexplained weight loss. Physical examination revealed an asthenic patient with mild mucocutaneous pallor, a temperature of 38°C, pulse rate of 110/min, blood pressure of 90/60 mmHg, and respiratory rate of 17/min. There was no clinical jaundice. Per abdominal examination revealed mild tenderness in the epigastric and right hypochondriac regions, with palpable liver extending one cm below the right subcostal margin, rounded margin. The spleen and superficial lymph nodes were not palpable. Initial investigations showed hemoglobin of 7.3 g/dl, total leukocyte count of 22,000 cells/mm^3^ (neutrophils 82%), and platelet counts (139,000 cells/mm^3^). Serum lactate dehydrogenase (LDH), C-reactive protein (CRP), and procalcitonin levels were elevated, but the levels of other tumor markers, such as alpha-fetoprotein (AFP) and carcinoembryonic antigen (CEA), were normal. Serology was negative for hepatitis B virus (HBV) surface antigen, HCV, and human immunodeficiency virus. An urgent noncontrast computed tomography (CT) examination of the abdomen and pelvis was performed which showed a heterogeneously hypodense lesion in the left lobe of the liver with multiple air foci within. The liver lesion was extending directly into the body of the stomach (Figure 1). The walls of the fundus and lesser curvature of the stomach could not be defined. These features were suggestive of rupture of left lobe liver abscess into the stomach. The spleen was remarkably normal, and the mesenteric, para-aortic, and retroperitoneal lymph nodes were not enlarged. Based on a presumptive diagnosis of liver abscess, the patient was initially treated with broad-spectrum antibiotics. The patient's clinical condition deteriorated, and a decision of surgical exploration was made. Intraoperative findings revealed dense adhesions of the left lobe of the liver with the anterior wall of the stomach and diaphragmatic surface. There was a 2 cm × 1 cm defect on the anterior wall of the stomach, and it was communicating with the abscess cavity in the left lobe of the liver. There was no peritoneal soiling. Necrosis tissues were removed from the liver, we drained the abscess cavity, and subhepatic drain was placed. The margins of the gastric perforation were freshened and primarily repaired (Figure 2). The patient was kept on nasojejunal tube for one week with broad-spectrum antibiotics and total parenteral nutrition. Histopathology of the surgical liver biopsy revealed a hepatic diffuse infiltration of large typical lymphoid B-cells, with necrosis. Immunohistochemistry was positive for CD20 and Ki-67 (80%) (Figure 3).

Postoperative investigations for disseminated non-Hodgkin lymphoma (NHL) by CT scan of the thorax did not reveal any lymphadenopathy or mass lesion. FDG-PET scan and bone marrow biopsy were negative. Thus, a diagnosis of primary non-Hodgkin lymphoma of the liver, large cell type, was confirmed. The patient condition did not allow additional complementary surgery, and he was managed with six cycles of R-CHOP regimen, rituximab, cyclophosphamide, doxorubicin, vincristine, and prednisone. However, a persistent fever occurred 1 month after three cycles of chemotherapy. The patient succumbed to neutropenic fever following chemotherapy.

## 3. Discussion

PHL and hepatogastric fistula are extremely rare. To our knowledge, this is the first report on a primary hepatic lymphoma invading the adjacent stomach, which was complicated with the development of a hepatogastric fistula. PHL was first described in 1965 by Ata and Kamel [1]. Some authors defined primary hepatic lymphoma as a very rare malignant tumor with the features of liver involvement and without involvement of other organs and tissues including the bone marrow, lymph nodes, spleen, and peripheral blood until at least 6 months after diagnosis [2, 3].

Primary hepatic lymphoma is very rare and constitutes about 0.0016% of all cases of non-Hodgkin's lymphoma (NHL) [4]. Extranodal lymphomas account for 10%–25% of non-Hodgkin's lymphomas, in which PHL is responsible for less than 1% [5].

PHL can occur at any age, and the average age of the reported patients is the fifth or sixth decade of life. It affects preferentially men with a male/female ratio of 2–3/1 [6]. PHL may be Hodgkin's or non-Hodgkin's; however, the latter is more common. Immunophenotypically, the B-cell is commoner than T-cell type [5]. The etiology of PHL is uncertain, and it may be associated with HIV, AIDS, hepatitis B and C, Epstein–Barr virus, liver cirrhosis, primary biliary cirrhosis, immunosuppressive therapy, and autoimmune diseases [7]. However, our case did not have any of the above conditions.

Symptoms are usually nonspecific and include hepatomegaly, gastrointestinal symptoms (abdominal pain, vomiting, and loss of appetite), right upper quadrant, and epigastric pain. Many cases are diffuse large B-cell lymphoma, and the patients show B-symptomatology of weight loss, fever, and night sweats, as well as fatigue and lethargy. Other rare clinical manifestations include pleural effusion, jaundice, thrombocytopenia, metabolic acidosis, and hypercalcemia [8]. Laboratory abnormalities associated with PHL include anaemia, neutropenia, hypercalcemia, and variably raised LDH, one serum alkaline phosphatase, b-microglobulin, and aminotransferase activities. The tumor markers AFP and CEA are found within normal range, in almost all cases of PHL [2].

On ultrasound imaging, majority are hypoechoic as compared to surrounding normal liver parenchyma. Radiological features of PHL are usually nonspecific, and the most common presentation on the computed tomography (CT) scan is a solitary hypoattenuating lesion, which may have a central area of low intensity indicating necrosis. Other less common radiological findings are multiple lesions and diffuse infiltration patterns [9]. On MRI, the lesion appears hypointense on T1-weighted and mildly hyperintense on T2-weighted images. However, it is often difficult to distinguish hepatic lymphoma from hepatocarcinoma or gastrointestinal tract metastasis because of the rarity of this disease and the nonspecific clinical presentation, laboratory, and radiologic features [10].

Diagnosis is often made upon histopathological and immunohistochemical investigation of the liver biopsy by using percutaneous needle aspiration, laparoscopy, or laparotomy [11]. In our case, liver biopsy was performed preoperatively because the lesion was profoundly symptomatic, and due to the rarity of the disease, we considered liver abscess with hepatogastric fistula in our first diagnosis.

Treatment modalities include surgical resection, chemotherapy, and radiotherapy alone or in combination. Most of the reported cases are diffuse large B-cell lymphomas, but this type is usually aggressive with a relatively poor prognosis [5]. Current literature favors combination chemotherapy as the frontline treatment owing to its noninvasive nature and improved survival outcomes. The standard treatment for patients with diffuse large B-cell lymphoma is CHOP. The addition of rituximab, a monoclonal antibody targeting the pan-B-cell antigenic marker CD20, to the CHOP regimen augments the complete response rate and prolongs overall survival [11, 12]. Optimal treatment is not yet defined. However, recent series reported favorable short and midterm outcomes and longer survival rates with the use of liver resection followed by adjuvant chemotherapy and/or radiation [13]. Surgery is a better option for localized disease with adequate normal liver volume, and preoperative chemotherapy can be tried with an attempt to reduce the tumor volume [5, 14]. However, because our patient's general condition did not permit, we did not proceed with a formal liver resection as indicated in this setting.

The clinical case we present is extremely unusual because there is a coincidence of two very rare features, primary hepatic lymphoma and hepatogastric fistula. Direct invasion to the gastric wall is the basis of the formation of a hepatogastric fistula. Nevertheless, a hepatogastric fistula is a rare complication, even more so for primary liver lymphoma. Such a fistula has been described following transarterial embolization, radiotherapy for hepatocellular carcinoma, percutaneous radiofrequency of hepatocellular carcinoma, pyogenic liver abscess, iatrogenic injury of the stomach, percutaneous catheter drainage of the liver abscess, or by direct infiltration of the stomach by hepatocellular carcinoma [15].

## 4. Conclusion

In conclusion, we have reported an exceptional case of PHL with an unusual complication that was difficult to discriminate from a liver abscess with a hepatogastric fistula. The preoperative diagnosis was difficult due to the rarity of the disease, the clinical and imaging manifestations of PHL were nonspecific, and levels of alpha-fetoprotein and carcinoembryonic antigen (CEA) were normal. The diagnosis of PHL should be strictly based on histopathology and immunophenotype. PHL should be considered in the differential diagnosis in a patient with space-occupying liver lesions. Favorable prognosis of PHL can be obtained by early surgery combined with chemotherapy in strictly selected patients.

## Data Availability

The research article data used to support the findings of this study are available from the corresponding author upon request (mbarekyaka@yahoo.fr).
